# Poultry red mite eradication potential of ivermectin and allicin combination treatment

**DOI:** 10.1002/vms3.1136

**Published:** 2023-04-05

**Authors:** JeongWoo Kang, HyunYoung Chae, Md Akil Hossain

**Affiliations:** ^1^ Animal Disease Diagnosis Division, Animal and Plant Quarantine Agency (APQA) Ministry of Agriculture Food and Rural Affairs Gimcheon‐si, Gyeongsangbuk‐do Republic of Korea; ^2^ Department of Oral Biology, College of Dentistry University of Illinois Chicago Chicago Illinois

**Keywords:** acaricides, asphyxiator, chloride channel, *Dermanyssus gallinae*, ectoparasite

## Abstract

**Background:**

Existing treatments against poultry red mite (PRM; *Dermanyssus gallinae*) infestation have reduced efficacy or exhibit hazardous effects on chickens. Considering the economic importance of chickens, development of a safe and effective method for exterminating PRMs is necessary. Ivermectin and allicin are effective against some ectoparasites; however, their acaricidal efficacies against PRMs remain unknown.

**Objective:**

To evaluate individual and combined efficacies of ivermectin and allicin in exterminating PRMs.

**Methods:**

Different concentrations (0.10–1.0 mg/mL) of ivermectin (1 mL) were applied via dropping method in different insect culture dishes (ICDs), prior to transferring PRMs. For the spraying method, PRMs were transferred to ICDs, before spraying ivermectin (1 mg/mL) solution (1 mL). Further, the acaricidal effect of allicin on PRMs was evaluated by applying different concentrations (0.25–1.0 mg/mL) of allicin (1 mL). The combined acaricidal effects of ivermectin and allicin were analysed using four concentration combinations. PRM death rates were determined after 2 h, 24 h, 2 days, 5 days and 7 days of drug application.

**Results:**

Ivermectin application (1 mg/mL) exterminated 64% and 100% of PRMs on 1 and 5 days, respectively, and prevented their revival. Further, 0.5 mg/mL ivermectin and 1 mg/mL allicin individually exterminated 98% and 44% of PRMs, respectively, within 7 days of treatment. In combination, 0.5 mg/mL ivermectin and 0.5 mg/mL allicin exterminated 100% of PRMs within 5 d of treatment. The most effective combination was 0.25 mg/mL ivermectin + 1.00 mg/mL allicin.

**Conclusions:**

The efficacy of ivermectin–allicin combination in exterminating PRMs was demonstrated. This novel approach could be optimised for industrial applications.

## Graphical abstract

Ivermectin and allicin synergistically exterminated poultry red mite (PRM; Dermanyssus gallinae) during 7 days of treatment, and this combination treatment may influence the physiological processes of PRMs.

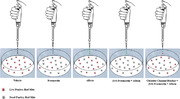

## 1 INTRODUCTION

*Dermanyssus gallinae*, generally known as poultry red mite (PRM), poultry mite, red mite, or chicken mite, is the most common and important blood‐sucking ectoparasite of layer and breeder flocks in many countries and probably the most widespread mite species found in birds in Europe (Abbas et al., 2014). PRMs attack resting chickens mainly at night for a short period (30–60 min) by sucking their blood (Kirkwood, 1968; Lancaster & Meisch, 1986). After the blood meal, PRMs take shelter in the crevices of the host skin, where they digest the sucked blood, mate, and lay their eggs (Abbas et al., 2014; Hearle, 1938). Nymphs and females suck blood, whereas males do so occasionally; larvae do not suck blood (Abbas et al., 2014). Severe PRM infestation may result in considerable blood loss (anaemia) and other diseases caused by transmitted viruses, bacteria and parasites (Abdel‐Ghaffar et al., 2009; Hungerford & Hart, 1937; Zeman et al., 1982), possibly leading to host mortality. Some of the other adverse effects of PRM infections in chickens are reduced egg quality due to blood spots, stressful behaviour and body weight reduction (Chauve, 1998). Therefore, PRM extermination is crucial for maintaining the good health of poultry and preventing gross agroeconomic losses.

Acaricide application is the main approach often employed to exterminate PRMs, and some acaricides have been approved globally for this purpose. The most widely used acaricides are organophosphates, carbamates, amidines and pyrethroid‐based acaricides. However, many acaricides are not recommended against PRMs and illegally used in poultry farms in various countries (Chauve, 1998; Beugnet et al., 1997; Marangi et al., 2009; Nordenfors et al., 2001; Zeman, 1987). For example, fipronil is an acaricide that is not classified as an ‘allowed substance’ for use as a veterinary medicinal product in food‐producing animals and birds (European Food Safety Authority (EFSA) 2018). Although some acaricides are effective against PRMs, they affect nontargets such as humans, poultry and eggs. Moreover, PRMs have developed resistance to different classes of acaricides in various regions worldwide. Therefore, several new alternative solutions, including biological compounds, essential oils, heat treatments, predator mites, inert dust, intermittent lighting programs and even vaccines, have been developed (Mozafar & Tierzucht, 2014). Furthermore, the anthelmintic drug ivermectin (0.5% lotion) can kill head lice (a human ectoparasite) (Deeks et al., 2013). Garlic has a lethal effect on northern fowl mites (Birrenkott et al., 2000), and its main component believed to be responsible for its biological activity is allicin (Cho et al., 2006). Thus, we hypothesised that ivermectin and allicin may interfere with the viability of PRMs. Accordingly, we aimed to evaluate the individual and combined efficacies of ivermectin and allicin in exterminating PRMs.

## 2 MATERIALS AND METHODS

**FIGURE 1:**
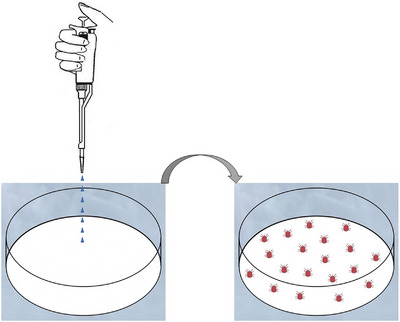
Schematic representation of the treatment method of the ivermectin–allicin combination to exterminate poultry red mites in this study.

Cardboard traps (100 mm × 70 mm × 3 mm) were installed in a poultry farm in Munkyeong City, Korea, to collect PRMs in accordance with a previously reported method (Chirico & Tauson, 2002); PRMs were collected in Ziploc plastic bags. A 90 mm filter paper (Advantech, Tokyo, 100‐0011, Japan) was placed in each insect culture dish (ICD), and 1 mL of different concentrations (5, 1, 0.5, 0.25, 0.1 and 0.05 mg/mL) of ivermectin solutions was applied to the filter papers in different ICDs by using a micropipette. On a working bench, the filter papers were left to soak the drug solution for 30 min and then partially dried (Figure 1). PRMs (20 individuals) were transferred to each ICD. The PRMs and the drug‐containing ICDs were stored in iceboxes, and the death rates of PRMs were recorded after 2 h, 24 h, 2 days, 5 days and 7 days of treatment.

For the spraying method, a 90 mm filter paper (Advantec, Tokyo, Japan) was kept in each ICD, and 20 PRMs were transferred to each of these ICDs. Approximately 1 mL (5 puffs) of ivermectin solution (1000 ppm) was sprayed onto the PRMs in the ICDs. The percentage of dead PRMs was determined after 2 h, 24 h, 2 days, 5 days and 7 days of ivermectin application.

Furthermore, the acaricidal effect of allicin on PRMs was evaluated by applying 1 mL of allicin (1, 0.5 and 0.25 mg/mL) with a micropipette. The percentage of dead PRMs after allicin treatment was determined using similar methods to ivermectin treatment. The combined acaricidal effects of ivermectin and allicin on PRMs were also analysed. Four combinations (0.25 mg/mL ivermectin + 0.25 mg/mL allicin; 0.25 mg/mL ivermectin + 0.5 mg/mL allicin; 0.25 mg/mL ivermectin + 1 mg/mL allicin; and 0.5 mg/mL ivermectin + 0.5 mg/mL allicin) were applied to 20 PRMs in ICDs by using a micropipette, and their effects were examined in accordance with the procedure described earlier in this section.

## 3 RESULTS AND DISCUSSION

Chemicals used to control PRMs may adversely affect workers through direct exposure and indirect consumption of pesticide residue‐containing eggs (Hamscher et al., 2003). We aimed to develop a more effective and convenient treatment for the successful extermination of PRMs without causing any harm to target/non‐target animals and humans by using ivermectin, which is an efficient and safe treatment for controlling the growth of PRMs (Zeman, 1987). In this study, we investigated the efficacy of ivermectin alone and in combination with allicin against PRMs. We applied 1 mg/mL ivermectin via two different methods, namely, dropping and spraying. We found that dropping was better than spraying, and the acaricidal ability of ivermectin and allicin gradually increased after treatment (Table 1). The combination of ivermectin and allicin showed synergistic and promising results, indicating that it can be used as a novel treatment option against PRMs.

**TABLE 1:** Individual and combination effects of ivermectin and allicin in the extermination of poultry red mites.

Sample (mg/mL)	Per cent (%) killing (mean ± standard deviation)				
	2 h	24 h	48 h	5 days	7 days
[Spray] ivermectin (1)	6.67 ± 2.89^*^	80.00 ± 26.46^*^	86.67 ± 23.09^*^	98.33 ± 2.89^*^	100.00 ± 0.00^*^
Ivermectin (1)	12.11 ± 8.55^*^	64.21 ± 15.39^*^	91.32 ± 8.95^*^	100.00 ± 0.00^*^	100.00 ± 0.00^*^
Ivermectin (0.5)	6.67 ± 5.77^*^	31.67 ± 24.66^*^	71.67 ± 12.58^*^	93.33 ± 2.89^*^	98.33 ± 2.89^*^
Ivermectin (0.25)	2 ± 2.74	26.00 ± 8.22^*^	54.00 ± 8.94^*^	77.00 ± 7.58^*^	87.00 ± 2.74^*^
Ivermectin (0.1)	1.67 ± 2.89	11.67 ± 12.58	26.67 ± 2.89^*^	36.67 ± 2.89^*^	40.00 ± 5.00^*^
Allicin (1)	15.00 ± 12.75^*^	23.00 ± 17.54	29.00 ± 12.94	35.00 ± 12.25	44.00 ± 9.62
Allicin (0.5)	5.00 ± 5.77	11.67 ± 7.64	15.00 ± 5.00	25.00 ± 5.00	30.00 ± 5.00
Allicin (0.25)	1.67 ± 2.89	10.00 ± 5.00	13.33 ± 10.41	18.33 ± 7.64	21.67 ± 7.64
Ivermectin (0.5) + Allicin (0.5)	38.33 ± 5.77^*^	73.33 ± 7.64^*^	90.00 ± 5.00^*^	100.00 ± 0.00^*^	100.00 ± 0.00^*^
Ivermectin (0.25) + Allicin (0.25)	3.33 ± 2.89^*^	31.67 ± 10.41^*^	51.67 ± 10.41^*^	90.00 ± 8.66^*^	93.33 ±2.89^*^
Ivermectin (0.25) + allicin (0.5)	3.33 ± 5.77	25.00 ± 13.23^*^	55.00 ± 10.00^*^	73.33 ± 5.77^*^	93.33 ± 2.89^*^
Ivermectin (0.25) + allicin (1)	11.67 ± 7.64^*^	58.33 ± 18.93^*^	80.00 ± 18.03^*^	91.67 ± 10.41^*^	98.33 ± 2.89^*^
Solvent control (10% ethanol)	0.00 ± 0.00	7.50 ± 5.46	11.43 ± 7.70	17.50 ± 11.05	20.00 ± 12.09
Untreated control	3.33 ± 5.77	5.00 ± 5.00	6.67 ± 2.89	11.67 ± 2.89	13.33 ± 2.89

Values are means ± standard deviation of 3 samples. *Statistical significance (*p* < 0.05) among solvent control and treated samples.

A significant number of PRMs (64%) were inhibited within 1 day of 1 mg/mL ivermectin application. The remaining PRMs were inhibited (100%) within 5 days of treatment (1 mg/mL). Ivermectin treatment also prevented PRM revival. Even 0.5 mg/mL ivermectin exterminated 32%, 72%, 93% and 98% of the PRMs after 1, 2, 5 and 7 days of treatment, respectively. Compared with the untreated control, lower ivermectin concentrations (0.25 and 0.1 mg/mL) showed considerable and concentration‐dependent extermination for different durations. Allicin (1 mg/mL) alone exterminated 23%, 29%, 35% and 44% of the PRMs after 1, 2, 5 and 7 days of treatment, respectively. However, lower allicin concentrations (0.5 and 0.25 mg/mL) exhibited relatively low PRM extermination rates. The number of PRMs remained stable until 2 days of observation although 11.67% and 13.33% of PRMs died on the fifth and seventh days of observation, respectively, under usual environmental conditions without any treatment. The diluent used to prepare the drug solution also elicited no remarkable effect on the mortality of PRMs. Within 24 h of treatment with the diluent, 7.50% of PRMs were exterminated, and 17.50% and 20.00% were exterminated on the fifth and seventh days of treatment, respectively.

The application of the ivermectin–allicin combination increased the mortality of PRMs. Different concentrations of the ivermectin–allicin combination were applied, and the acaricidal effects of some combinations, which showed synergistic or additive effects are stated in this report. The acaricidal effect of the combination of 0.5 mg/mL ivermectin and 0.5 mg/mL allicin was greater than that of individual drugs used at the same concentrations and other drug combinations. The PRMs were completely exterminated on the fifth day of treatment with 0.5 mg/mL ivermectin and 0.5 mg/mL allicin. The effects of both drugs were concentration and exposure‐duration dependent, and ivermectin was more potent than allicin. However, a high ivermectin concentration can be retained in poultry eggs. Therefore, it is prohibited in many countries for food safety. In this study, we evaluated the effects of low concentrations of ivermectin, which may have no adverse effects to nontargets and consumers if it retains in poultry eggs and meat. Although high allicin concentration (30–59 mg) may have mild adverse effect such as stomach irritation, but the application of allicin table (5 mg), and injection (1 mg) could not show adverse effect to human suggesting that allicin is safe (Salehi et al., 2019). The combination of ivermectin and allicin showed synergistic and promising results, which indicated that it can be used as a novel treatment option against PRMs. Ivermectin can efficiently kill human head lice, a parasite with physiological properties similar to those of PRMs (Chosidow et al., 2010). Ivermectin targets glutamate‐gated chloride channel receptors found on neurons and muscle cells of organisms, causing paralysis and death (Atif et al., 2017). The potential efficacy of ivermectin in this study suggested that it may affect vital physiological processes of PRMs, such as the functioning of chloride channels (Table 2). It is reported that the exposure of bugs to allicin causes suffocation and death of them. Similarly, allicin application causes suffocation of insects followed by interfering with the neurotransmitter receptors in their nervous systems (Hazafa et al., 2021). Similar as in insects and bugs, allicin may affect the neurotransmitter receptors in PRMs. Picrotixin, a chloride channel blocker, slightly decreased the killing effect of ivermectin and allicin combination. Similar to ivermectin alone, the ivermectin–allicin combination may influence the physiological processes of PRMs. However, further studies are required to determine the specific mechanism of PRM extermination.

**TABLE 2:** Effectiveness of ivermectin in the presence of chloride channel blockers (picrotoxin) in the extermination of poultry red mites.

Sample (mg/mL)	Per cent (%) of killing (mean ± SD)		
	2 h	24 h	48 h
I 0.25 + A 1	10.00 ± 13.23	60.00 ± 13.23^*^	81.00 ± 5.00^*^
P 1	3.33 ± 2.89	10.00 ± 5.00	13.33 ± 2.89
I 0.25 + A 1 + P 1	6.67 ± 7.64	36.67 ± 11.30	55.00 ± 5.00
Solvent control	1.67 ± 2.89	5.45 ± 2.89	6.79 ± 3.55

Values are means ± standard deviation of 3 samples. *Statistical significance (*p* < 0.05) between I0.25+A1+P1 and I0.25+A1. I, ivermectin; A, alicin; P, picrotoxin.

Our findings demonstrated that the combination of 0.25 mg/mL ivermectin and 1.00 mg/mL allicin could exterminate PRMs and could be optimised for clinical use. Nevertheless, the development of a suitable dosage of 0.25 mg/mL ivermectin and 1.00 mg/mL allicin combination would be effective for its rapid application and transport.

## AUTHOR CONTRIBUTIONS

Conceptualisation, J.W.K., M.A.H. Methodology, J.W.K., M.A.H. Investigation, J.W.K., M.A.H. Resources, J.W.K. Data curation, H.Y.C, M.A.H. Writing – original draft preparation, J.W.K. Writing, review and editing, J.W.K., M.A.H. Supervision, J.W.K. Project administration, J.W.K. Funding acquisition, J.W.K. All authors have read and agreed to the published version of the manuscript.

## FUNDING INFORMATION

This study was supported by veterinary science research project grants from the Animal and Plant Quarantine Agency, Ministry of Agriculture, Food and Rural Affairs, Republic of Korea.

## CONFLICT OF INTEREST STATEMENT

The authors declare no conflicts of interest.

### PEER REVIEW

The peer review history for this article is available at https://publons.com/publon/10.1002/vms3.1136.

## Data Availability

All data of this study are included in the manuscript.
